# Role of *Callistemon citrinus* Leaf Phytosomes Against Oxidative Stress and Inflammation in Rats Fed with a High-Fat-Fructose Diet

**DOI:** 10.3390/antiox13101263

**Published:** 2024-10-18

**Authors:** Luis Gerardo Ortega-Pérez, José Armando Hernández-Soto, Osvaldo Padilla-Avalos, Luis Alberto Ayala-Ruiz, Oliver Rafid Magaña-Rodríguez, Jonathan Saúl Piñón-Simental, Asdrúbal Aguilera-Méndez, Daniel Godínez-Hernández, Patricia Rios-Chavez

**Affiliations:** 1 Facultad de Biología, Universidad Michoacana de San Nicolás de Hidalgo (UMSNH), Morelia 58000, Michoacán, Mexico; gerardo.ortega@umich.mx (L.G.O.-P.); 1719780c@umich.mx (J.A.H.-S.); 1900289a@umich.mx (O.P.-A.); 1232816g@umich.mx (L.A.A.-R.); oliver.rodriguez@umich.mx (O.R.M.-R.); 1434491c@umich.mx (J.S.P.-S.); 2 Instituto de Investigaciones Químico Biológicas, Universidad Michoacana de San Nicolás de Hidalgo (UMSNH), Morelia 58000, Michoacán, Mexico; amendez@umich.mx (A.A.-M.); daniel.godinez@umich.mx (D.G.-H.)

**Keywords:** *Callistemon citrinus*, anti-inflammatory, antioxidant

## Abstract

Phytosomes are used as vehicles that carry plant extracts. They exhibit biological activities and possess better bioavailability, bioabsorption, and lower toxicity than drugs. Obesity is an inflammatory state in which oxidative stress is present, which triggers severe effects on the body’s organs. This study aimed to evaluate the impact of the extract and phytosomes of *Callistemon citrinus* on oxidative stress and inflammation in the liver and heart of Wistar rats fed with a high-fat-fructose diet. Phytosomes containing the extract of leaves of *C. citrinus* were prepared. The antioxidant, pro-inflammatory enzymes, and biomarkers of oxidative stress were evaluated. Among the groups, only the high-fat-fructose group presented an increase in the COX-2, 5-LOX, and MPO inflammatory enzymes, while the XO enzyme exhibited decreased activity. The groups were fed a hypercaloric diet for 15 weeks while orlistat, *C. citrinus* extract, and phytosomes were administered at three different concentrations, exhibiting enzyme activities similar to those of the control group. It was also observed that the lowest concentration of phytosomes had a comparable effect to the other concentrations. *Callistemon citrinus* extract can modulate the activities of enzymes involved in the inflammation process. Furthermore, small doses of phytosomes can serve as anti-inflammatory agents.

## 1. Introduction

Obesity is a complex disease characterized by the excessive accumulation of body fat, which can be detrimental to health. It leads to an increase in inflammatory markers, causing chronic low-grade inflammation. Additionally, obesity increases the risk of developing type 2 diabetes and cardiovascular diseases, negatively affects bone health and reproduction, and raises the risk of certain cancers [1].

A high-fat-sugar diet can lead to obesity, activating innate immune cells and triggering chronic inflammation, especially in adipose tissue and the liver [2]. Obesity produces reactive oxygen species (ROS) [3]. The primary sources of ROS are the mitochondrial respiratory chain and nicotinamide adenine dinucleotide phosphate oxidase (NADPH oxidase/NOX), which are byproducts of cellular metabolism. There is an enzymatic antioxidant defense system that includes superoxide dismutase (SOD), catalase (CAT), and glutathione peroxidase (GPx). Additionally, non-enzymatic antioxidants like glutathione, thioredoxin, and vitamins C and E also protect cells from reactive oxygen species (ROS) by scavenging or neutralizing them. When there is an imbalance between ROS and the antioxidant system, oxidative stress is induced. It is implicated in many diseases, including diabetes mellitus, atherosclerosis, and stroke. These oxidants attack lipids, proteins, sugar, and nucleic acid, producing molecules that can be used as oxidative biomarkers and lipid peroxidation products like malondialdehyde (MDA) and 4-hydroxynonenal (HNE) [4] and advanced oxidation protein products (AOPPs) [5].

Plant-based compounds may help reduce intracellular oxidative stress in obesity. Various plant-based foods are effective for weight management. Therefore, a balanced diet should consistently include adequate fruits, vegetables, spices, and herbs [6]. They contain various compounds, such as terpenes, phenolics, and flavonoids, which have antioxidant, antitumor, and anti-inflammatory properties. As a result, they can be used as a natural option for treating various chronic diseases in which inflammation and oxidative stress are important factors [7].

The *Callistemon* genus is a promising group of medicinal plants known for their neuroprotective, chemopreventive, antioxidative, anti-aging, antimicrobial, and other pharmaceutical properties [8,9]. *Callistemon citrinus* (Curtis) Skeels is cultivated for its ornamental beauty and is also utilized by traditional healers in crafting herbal medicinal formulations [10]. In a previous study by Petronilho et al. [11], the phytochemical characterization of *Callistemon citrinus* was reported. Over 77 terpene compounds were found in the ethanolic extract of *C. citrinus* leaves, and they were identified using two-dimensional gas chromatography with time-of-flight mass spectrometry detection (GC x GC-ToFMS). The main compounds identified were 1,8-cineole, limonene, and α-terpineol. Additionally, Ortega-Pérez et al. [12] reported the presence of gallic acid, p-coumaric acid, and ellagic acid using HPLC. Recent studies suggest that *C*. *citrinus* has a significant anti-obesogenic effect through mechanisms that include anti-lipase, antioxidant, and anti-inflammatory activities [9,12,13] and the modulation of oxidative stress [14].

Phytosomes are a delivery system for drugs or plant extracts formulated with phospholipids [15]. This method stands out from conventional drug or plant extract delivery methods by providing a more stable profile. It offers resistance against degradation by digestive enzymes and microbiota, enhances membrane permeability, increases bioavailability, and amplifies the effectiveness of the phytoconstituents [16]. There is limited research on using phytosomal formulations in anti-obesity drugs. Developing antioxidant and anti-inflammatory herbal drugs in phytosomal forms is essential to improving bioavailability and reducing adverse effects. Embracing the phytosomal approach in drug delivery could help overcome the limitations of traditional drug delivery methods [17]. The utilization of *Callistemon citrinus* phytosomes has shown promising potential in controlling weight in rodent models of obesity, presenting an innovative approach for utilizing *C. citrinus* in obesity treatment [12]. This research aims to assess the effectiveness of *C. citrinus* leaf phytosomes in regulating oxidative stress and inflammation in male Wistar rats fed a hypercaloric diet.

## 2. Materials and Methods

### 2.1. Chemicals

5,5-dithiobis-2-nitrobenzoic acid, blue nitrotetrazolium chloride, 1-chloro-2,4-dinitrobenzene, 4-nitrophenyl acetate,3,3′,5,5′-tetramethylbenzidine, N,N′,N″,N‴-tetramethyl-p-phenylenediamine and all other chemicals and reagents were of analytical grade and purchase from Sigma-Aldrich Company, Ciudad de México, México.

### 2.2. Plant Material and Preparation of Ethanolic Extract

To ensure the highest compound content and antioxidant effectiveness of the leaf extract [14], leaves of four-year-old *Callistemon citrinus* (Curtis) Skeels (Myrtaceae) were collected in Morelia, Michoacán, Mexico. The leaves were macerated in 96% ethanol (1:10 *w*/*v*) at room temperature for five days. Following maceration, the extract was concentrated using a rotary evaporator set at 45 °C. Standardization was made as specified by López-Mejía [14]. Our previous study [12] contains information about phytosome preparation.

### 2.3. Animals

Male Wistar rats, 2 months old and weighing 180–200 g, were obtained from the animal laboratory of Instituto de Investigaciones Químico Biológicas at UMSNH. The animals were kept in plastic enclosures in a controlled environment with a 12 h light-dark cycle, humidity levels maintained between 60 and 70%, and temperatures kept at 20–24 °C. Food and water were available at all times. These animals were maintained in the bioterium of the Instituto de Investigaciones Químico Biológicas at UMSNH. All experimental procedures were approved and adhered to the laboratory animal care guidelines of UMSNH Ethics Committee (approval date: 01/12/2023; protocol ID IIQB-CIBE-06-2023) and the established by the Official Mexican Norm (NOM-062-ZOO-1999) of Mexican secretary of Agriculture, livestock, rural development, fishing and food. Official Diary, Ciudad de Mexico, México, 2001.

### 2.4. Induction of Obesity by Feeding High-Fat-Fructose Diet

Obesity has been investigated in various animal models due to the consumption of high-calorie diets and the presence of oxidative stress [18]. A high-fat diet (HFD) comprised of 45.4% standard chow (Rodent diet brand: Purina^®^ rat chow), 14.8% lard, 14.8% vegetable fat, and 25% fructose was prepared daily, as detailed in Ortega-Pérez et al. [12]. Fifty-four male Wistar rats were randomly divided into nine groups (*n* = 6). Group 1 received a chow diet; Group 2, a chow diet plus vehicle; Group 3, a chow diet plus *C. citrinus* extract (200 mg/kg); Group 4, HFD; Group 5, HFD plus orlistat (5 mg/kg); Group 6, HFD plus *C. citrinus* extract (200 mg/kg); Group 7, HFD plus phytosomes loaded with *C. citrinus* (P *C. c*.) (50 mg/kg); Group 8, HFD plus P *C. c*. (100 mg/kg) and Group 9, HFD plus P *C. c.* (200 mg/kg). Treatments were administered by oral gavage once daily at 9:00 a.m. for 15 weeks. Daily food and water intake measurements were taken, and the animals’ weight was recorded every week to calculate the final body weight gain after the experimental model. The animals’ age at the end of the treatment was 23 weeks. Blood samples were collected by cardiac puncture after 12–13 h of fasting. Then, the animals were anesthetized using an intraperitoneal injection of pentobarbital sodium at 150 mg/kg, and all tissues were harvested, washed, and stored at −80 °C for subsequent analysis.

### 2.5. Tissue Preparations

Liver and heart tissues weighing 0.25 g were homogenized in 1 mL of a 50 mM phosphate buffer at pH 7.4 with 0.1 M EDTA. Subsequently, they were centrifuged using a centrifuge 5424 R Eppendorf at 13,000 rpm for 20 min at 4 °C. The resulting supernatant was collected, frozen, and stored at −80 °C for further biochemical enzymatic and biomarker estimation. The protein concentration in all the homogenates was determined using Bradford’s method, using a UV Vis Spectrophotometer genesis 50 [19].

### 2.6. Total Oxidative Status (TOS) Assay

The ferrous ion–o-dianisidine complex is oxidized to a ferric ion. At acidic pH levels, this procedure results in the formation of a colored complex with xylenol orange. Reagent 1 contained 150 μM of xylenol orange, 140 mM of NaCl, and 1.35 M of glycerol at pH 1.75. Reagent 2 contained 5 mM of ferrous ammonium sulfate and 10 mM of o-dianisidine dihydrochloride. Briefly, 450 μL of reagent 1, 70 μL of the sample, and 422 μL of reagent 2 were mixed. Absorbance readings were taken at two points: the first immediately before the combination of reagents 1 and 2 to serve as the sample blank, and the final reading when the reaction’s progress flattens into a plateau, approximately 3–4 min post-mixing, indicating the end-point measurement. The assay is standardized using hydrogen peroxide, and results are presented as μmol H_2_O_2_ Equiv./L. [20].

### 2.7. Estimation of Malondialdehyde (MDA) and Hydroxyalkenals (HNE)

MDA levels were determined in a mixture consisting of 200 μL of homogenate (liver or heart), 5 μL of 5 mM butylated hydroxytoluene (BHT), 650 μL of 10 mM 1-methyl-2-phenylindole, and 150 μL of 37% HCl. The reaction mixture was incubated at 45 °C for 60 min, then cooled on ice to stop the reaction and measured at 586 nm. The total HNE plus MDA assay was conducted similarly but with 37% methane sulfonic acid instead of hydrochloric acid, the blank without the sample. The levels of lipid peroxidation products were expressed in nmol MDA/g tissue and nmol HNE/g tissue [21].

### 2.8. Advanced Oxidation Protein Products Level (AOPP)

In brief, 50 μL of homogenate was mixed with 1 mL of 20 mM phosphate buffer at pH 7.4, followed by adding 50 μL of 1.16 M potassium iodide and 100 μL of acetic acid. After a 2 min wait, the absorbance was measured at 340 nm. The standard curve for chloramine-T ranged from 0 to 100 μmol/mL. The concentration of AOPP was reported in μmol/mL of chloramine-T equivalents [22].

### 2.9. Reduced Glutathione (GSH) Levels

The assay mixture comprised 62.5 μL of homogenate, 187.5 μL of 0.2 M phosphate buffer at pH 8.2, and 12.5 μL of 0.01 M 5,5-dithiobis-2-nitrobenzoic acid (DTNB). Following this, 987.5 μL of absolute methanol was added. The mixture was then placed in a laboratory mixer and agitated at 240 rpm for 15 min. After agitation, it was centrifuged at 1250 g for 15 min at room temperature. This process resulted in the development of a yellow color, and the absorbance was measured at 412 nm. The results are reported in units of mM GSH per gram of tissue [23].

### 2.10. SOD Activity 

When exposed to light, superoxide dismutase (SOD) interacts with superoxide anions produced by riboflavin, thereby slowing down the formation of the formazan product. The assay mixture comprised 10 μL of homogenate (from the liver or heart), 641 μL of 0.067 M phosphate buffer at pH 7.0, 40 μL of 0.1 M EDTA, 20 μL of 1.5 mM blue nitrotetrazolium chloride (NTB), and 9 μL of 0.1 mM riboflavin. After gentle stirring, the mixture was illuminated from a distance of 15 cm using a 40-watt lamp for 15 min; the absorbance was measured at 560 nm. All components except the tissue sample were included in the blank, which was not illuminated. The specific activity of the enzyme was measured in U/mg protein. One unit of SOD is defined as the amount of enzyme required to achieve a 50% inhibition of the NBT reduction rate within one minute at room temperature [24].

### 2.11. CAT Activity 

The catalase (CAT) activity was measured based on the rate at which hydrogen peroxide (H_2_O_2_) decomposed. The assay mixture consisted of 950 μL of 50 mM phosphate buffer at pH 7.0, 25 μL of homogenate (from liver and heart), and 25 μL of 30 mM H_2_O_2_ as the substrate. The change in absorbance at 240 nm was recorded every 30 s for three minutes. The enzyme-specific activity was expressed in µmol H_2_O_2_ consumed per minute per mg of protein, based on a molar extinction coefficient of 43.6 M^−1^ cm^−1^ [25].

### 2.12. GPx Activity 

The working solution, totaling 975 μL, consisted of 0.5 mM NADPH, 100 mM reduced glutathione, 1 unit of the enzyme glutathione reductase, 50 mM phosphate buffer at pH 7.0, 30 mM cumene hydroperoxide, and 25 μL of homogenate from the liver and heart. Following stirring, the absorbance at 340 nm was measured every 30 s for five minutes. The activity of glutathione peroxidase (GPx) was defined as one unit per minute per mg of protein, based on the oxidation of one nmol of NADPH, using the molar extinction coefficient of 6.22 mM^−1^ cm^−1^ [26].

### 2.13. GST Activity

A 999 μL working solution was prepared by combining 980 μL of 0.1 M phosphate buffer at pH 6.5, 10 μL of 100 mM 1-chloro-2,4-dinitrobenzene (CDNB) as the substrate, and 10 μL of 10 mM reduced glutathione. This solution was then incubated at 30 °C for 15 min. After incubation, 1 μL of homogenate from the liver and heart was added. The absorbance at 340 nm was measured every 30 s for five minutes. Glutathione-S-transferase (GST) activity was calculated as the amount of GSH-CDNB conjugate formed per minute per mg of protein (min/mg protein), using a molar extinction coefficient of 9.6 mM^−1^ cm^−1^ [14].

### 2.14. PON1 Activity 

4-nitrophenyl acetate was employed to assess paraoxanase-1 (PON1) activity. A mixture comprising 5 μL of homogenate and 1 mL of working reagent, consisting of 25 mM Tris buffer at pH 8.0, 10 mM CaCl2, and 1 mM 4-nitrophenyl acetate, was prepared. The absorbance at 402 nm was monitored every 30 s for three minutes, with water serving as the blank to correct for non-enzymatic hydrolysis. PON1 activity was quantified as the production of 1 µM of phenol per minute per mg of protein, using the molar extinction coefficient of 14,000 M^−1^ cm^−1^ [27].

### 2.15. MPO Activity

Homogenates were prepared from 0.1 mg of liver or heart tissue in 50 mM phosphate buffer (pH 7.4) containing 0.5% hexadecyl-trimethyl ammonium bromide. After sonication for 15 s, the samples underwent three freeze–thaw cycles and were centrifuged at 17,000 rpm for 20 min at 4 °C. The supernatant obtained was used to measure myeloperoxidase (MPO) levels. The reaction mixture was composed of 425 µL of 200 mM phosphate buffer (pH 5.4), 10 µL of 15 mM H_2_O_2_, and 40 µL of 20 mM 3,3′,5,5′-tetramethylbenzidine (TMB). Subsequently, 25 µL of the supernatant was added. The mixture was then incubated at 37 °C for 3 min in darkness, followed by a 3 min incubation on ice. To halt the reaction, 1000 µL of 200 mM sodium acetate (pH 3) was added, and the absorbance was measured at 655 nm for 3 min [28].

### 2.16. COX-1 and COX-2 Activity

The peroxidase activity of cyclooxygenase (COX-1, COX-2) was assessed using the method outlined by Kumar et al. [29]. In brief, the reaction mixture comprised 712 µL of 100 mM Tris-HCl buffer (pH 8), 31 µL of 15 µM hematin, 31 µL of 3 µM EDTA, 100 µL of liver or heart homogenate, and 63 µL of 100 mM N,N′,N″,N‴-tetramethyl-p-phenylenediamine (TMPD). Subsequently, 63 µL of 133 µM arachidonic acid was introduced as a substrate, and the mixture was incubated for 20 min at 25 °C. The absorbance was then measured at 590 nm. Concurrently, to differentiate COX-1 activity, a tube was prepared for each sample containing an inhibitory substrate (etoricoxib, a selective COX-2 inhibitor). The extinction coefficient for TMPD was 0.00826 µM^−1^. Enzyme activity was defined as the amount required to oxidize 1 nmol of TMPD per minute.

### 2.17. 5-LOX Activity

The 5-lipoxygenase (5-LOX) assay relies on the formation of the Fe^3+^/xylenol orange salt complex. The reaction mixture comprises 490 µL of 50 mM Tris-HCl buffer at pH 7.4, 10 µL of sample homogenate, and 10 µL of 133 µM arachidonic acid. This mixture is then thoroughly mixed and left to incubate at room temperature in darkness for 10 min. Subsequently, 490 µL of FOX reagent is introduced. The FOX reagent is composed of 25 mM sulfuric acid, 100 µM xylenol orange, and 250 µM ferrous sulfate, diluted in water-methanol (1:9). Additionally, 100 µL of 4 mM butylhydroxytoluene is included as an antioxidant to prevent lipid oxidation. The solution is mixed and incubated as previously described. Finally, the absorbance at 590 nm is measured [29].

### 2.18. XO Activity

Blair’s method [30] was used to assess xanthine oxidase (XO) activity. The assay mixture comprised 1000 µL of 33 mM phosphate buffer at pH 7.5, 500 µL of sample homogenate, and 100 µL of 0.17 mM xanthine. The reaction proceeded by incubating the mixture at 37 °C for 1 h. Subsequently, 100 µL of 100% trichloroacetic acid was added, followed by centrifugation at 10,000× *g* for 15 min.

The uric acid was quantified in the transparent supernatants, and the absorbance was measured at 293 nm. Control blanks had the same composition as the reaction mixture without xanthine. Enzyme activity was determined by comparing the reaction rates with and without xanthine. Xanthine oxidase activity was measured in µmoles of uric acid generated per hour per gram of wet tissue weight. It was expressed as specific activity in µmoles per hour per milligram of protein.

### 2.19. Statistical Analysis

The test results were presented as the mean ± standard error (SEM) or standard deviation (SD). Data were analyzed using GraphPad Prism (version 8.0) with a one-way analysis of variance (ANOVA). Tukey’s multiple comparison test was used to identify statistical differences (a, b, c) in biomarkers and enzymatic parameters between groups. A result of * *p* ≤ 0.05 is considered statistically significant. Tukey’s Honestly Significant Difference (HSD) test is a post hoc test used in ANOVA to compare all possible pairs of means. When a significant difference among group means is found through ANOVA, a post hoc test like Tukey’s is essential to determine which group is significantly different.

## 3. Results

### 3.1. Effect of Phytosomes of C. citrinus on Morphometric Parameters and Serum Total Oxidative Status (TOS) of High-Fat-Fructose-Fed Rats

It was observed that rats fed a high-fat-fructose diet experienced weight gain, resulting in increased adipose tissue. In contrast, rats treated with orlistat showed almost 50% reduction in adipose tissue compared to the diet-only group. Treatment with *C. citrinus* extract and phytosomes significantly decreased adipose tissue, similar to the control group (Table 1).

**Figure 1:**
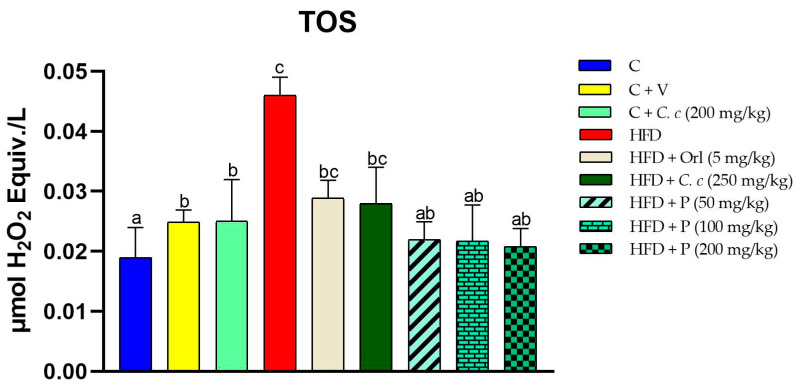
Total Oxidative Status (TOS) in various treatment groups. Values are expressed as mean ± standard error (ANOVA followed by Tukey’s test, *n* = 6). Different letters (a, b, c) indicate statistically significant group differences.

The rats fed a high-fat-fructose diet demonstrated a notably higher increase in TOS levels than the other groups. The control group exhibited the lowest TOS levels. Moreover, supplementation with *C. citrinus* at 200 mg/kg did not significantly affect TOS levels compared to the control group, indicating that *C. citrinus* does not induce oxidative stress independently. This result aligns with that of the vehicle group. The administration of *C. citrinus* extract and phytosomes resulted in significant decreases in TOS levels compared to the HFD group. Notably, the phytosomes at 50 mg/kg demonstrated an equivalent effect to those at 200 mg/kg and the *C. citrinus extract* (Figure 1).

### 3.2. Antioxidant Enzyme Activities

**Figure 2:**
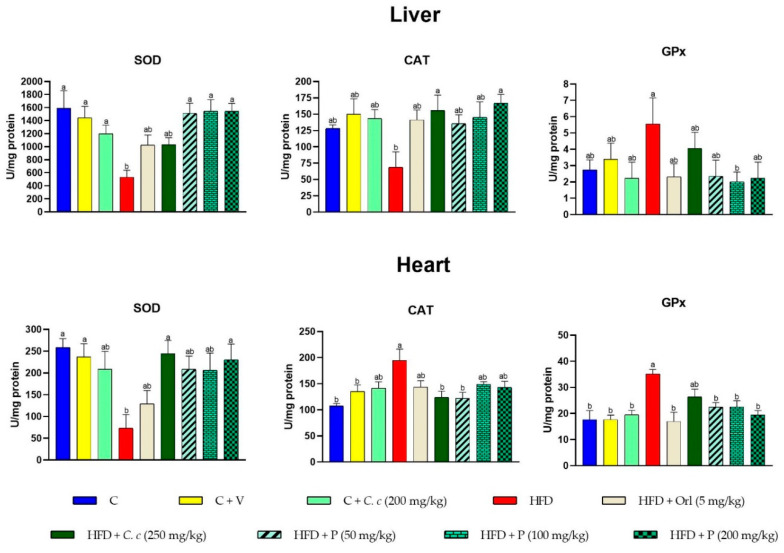
Effect of *C. citrinus* extract and phytosomes on the superoxide dismutase (SOD), catalase (CAT), and glutathione peroxidase (GPx) activities in liver and heart tissues. Control (C), Control + Vehicle (C + V), Control + *C. citrinus* extract (C + *C. c*, 200 mg/kg), High-Fat Diet (HFD), HFD + Orlistat (HFD + Orl, 5 mg/kg), HFD + *C. citrinus* extract (HFD + *C. c*., 250 mg/kg), HFD + Phytosome (HFD + P, 50 mg/kg, 100 mg/kg, and 200 mg/kg, respectively). Values are expressed as mean ± standard error (ANOVA followed by Tukey’s test, *n* = 6). Different letters (a, b) indicate statistically significant differences between groups.

As illustrated in Figure 2, the SOD and CAT activities in the liver were lower in the HFD group compared to all other groups. However, when *C. citrinus* phytosomes were administered at three different doses, the SOD and CAT activities in the liver increased by 50% compared to the HFD group. Additionally, the administration of 200 mg/kg of *C. citrinus* phytosomes and Cc extract at 250 mg/kg resulted in CAT activity levels higher than those in the control group. This same pattern was observed in the SOD activity in the heart. On the other hand, CAT activity in the heart and GPx in the liver and heart showed an increase in the HFD group, unlike the other groups. Treatments with extracts and phytosomes exhibited protective potential, restoring or maintaining the activity of these enzymes at levels similar to the control. These findings are relevant for developing antioxidant-based therapeutic strategies to mitigate the effects of oxidative stress induced by a high-fat diet.

**Figure 3:**
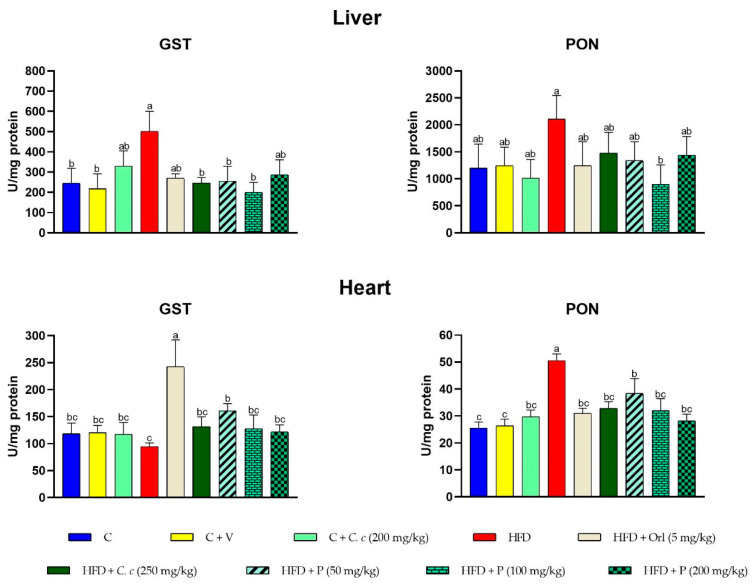
Effect of *C. citrinus* extract and phytosomes in glutathione S-transferase (GST) and paraoxonase-1 (PON1) in liver and heart tissues. Values are expressed as mean ± standard error (ANOVA followed by Tukey’s test, *n* = 6). Different letters (a, b, c) indicate statistically significant group differences.

The rats fed a high-fat-fructose diet exhibited significantly higher GST activity in the liver and lower activity in the heart. Additionally, PON1 activity increased in both tissues in the HFD group. The supplementation of *C. citrinus* extract and phytosomes with the high-fat diet helped to maintain GST and PON activities in both tissues, similar to the control group (Figure 3).

### 3.3. Biomarkers of Oxidative Stress

Oxidative damage in the liver and heart was assessed using markers such as MDA, HNE, AOPP, and reduced glutathione (GSH). The results revealed a significant increase in MDA, HNE, and AOPP levels in the liver and heart of the high-fat diet (HFD) group, which showed a negative correlation with GSH levels in both tissues. However, the treatment with *C. citrinus* extract and three doses of phytosomes showed a protective effect by reducing MDA, HNE, and AOPP levels and increasing GSH levels in these tissues, similar to the control group (Table 2).

### 3.4. Pro-Inflammatory Enzymes Activities

**Figure 4:**
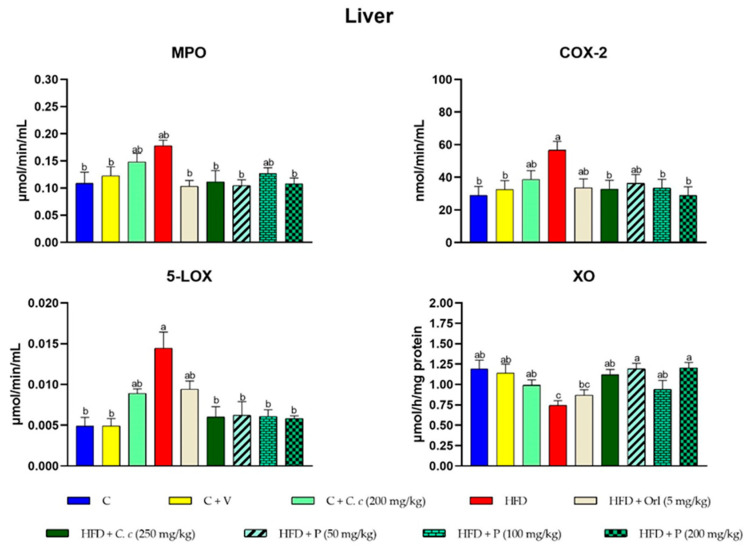
Effects of different treatments on the enzymatic activities of MPO, COX-2, 5-LOX, and XO in liver tissue. Values are expressed as mean ± standard error (ANOVA followed by Tukey’s test, *n* = 6). Different letters (a, b, c) indicate statistically significant group differences.

**Figure 5:**
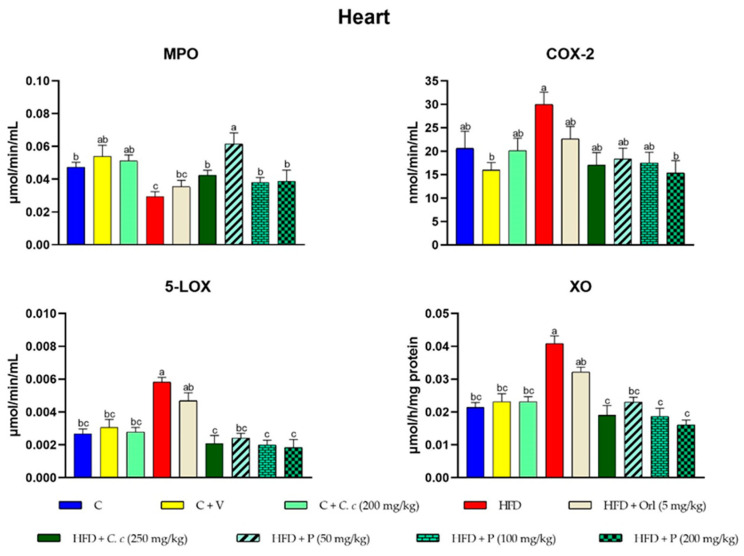
Effects of different treatments on the enzymatic activities of MPO, COX-2, 5-LOX, and XO in heart tissue. Values are expressed as mean ± standard error (ANOVA followed by Tukey’s test, *n* = 6). Different letters (a, b, c) indicate statistically significant group differences.

The results were further substantiated by examining the inflammatory activities, as shown in Figure 4. Myeloperoxidase (MPO), cyclooxygenase-2 (COX-2), and 5-lipoxygenase (5-LOX) activities were notably elevated in the livers of rats subjected to a high fat-fructose diet (HFD) compared to both the control group and the groups receiving *C. citrinus* extract and phytosomes supplementation. Conversely, xanthine oxidase (XO) activity was reduced in the HFD group (Figure 4). In heart tissue, COX-2, 5-LOX, and XO activities were higher in the HFD group, while MPO activity decreased compared to the control group (Figure 5).

## 4. Discussion

The study demonstrated that supplementing rats with *Callistemon citrinus* extract and phytosomes while feeding them a high-fat-fructose diet for 15 weeks reduced body weight gain and adipose tissue. Additionally, it improved antioxidant levels and decreased markers of oxidative stress and inflammation in the liver and heart. Importantly, even a low concentration of phytosomes (50 mg/kg) was sufficient to produce beneficial effects. Ortega-Pérez et al. [12] previously reported that treatment with phytosomes significantly decreased plasma triacylglycerol levels in HFFD-fed rats, leading to improved lipid metabolism and reduced fat accumulation. This was evidenced by the Adiposity index, which was 9.4 ± 0.62 in the high-fat-fructose-diet group and 4.02 ± 0.62 in the group treated with phytosomes at 200 mg/kg.

The protective effect of *C. citrinus* extract and phytosomes is attributed to its main compounds, including 1,8-cineole, limonene, and α-terpineol, as reported in *C. citrinus* [11]. In addition, gallic acid, p-coumaric acid, and ellagic acid [12] possess scavenging free radical activities, as shown by López-Mejía et al. [14]. Additionally, Ayala-Ruiz et al. [31] have reported that these three terpenes boost the activities of antioxidant enzymes such as SOD, CAT, and GPx. Furthermore, they reduce the oxidative stress biomarkers’ levels and lower the cytokines IL-6, TNF-α, and leptin concentrations in the livers of Wistar rats fed a high fat-sucrose diet for 15 weeks. Moreover, these terpenes demonstrate protective effects against the liver damage induced by a high-fat-sucrose diet. On the other hand, the *C. citrinus* extract and phytosomes exhibit gastroprotective properties by inhibiting anti-inflammatory enzymes MPO, COX-2, 5-LOX, and XO [13].

The *C. citrinus* extract and the phytosomes exhibit strong antioxidant activity against oxidative stress in rats fed with a high-fat-fructose diet. However, there are differences between them in terms of total oxidative status. The activities of SOD in the liver and GPx in the heart show that the three doses of phytosomes have better antioxidant capacity than the extract. Phytosomes (200 mg/kg) as the extract showed similar activities of SOD, GST, PON-1, MPO, XO, and 5-LOX in the heart, and CAT, GPx, PON-1, MPO, COX-2 in the liver. Concerning the levels of GSH and MDA in both tissues and the activity of 5-LOX in the liver, the three doses of phytosomes and the extract had the same values. An interesting finding was that the lowest levels of HNE and AOPP in both tissues were observed with the phytosomes at 200 mg/kg compared to the other two doses of phytosomes and the extract.

The effect of phytosomes at a dosage of 50 mg/kg was similar to those at dosages of 100 and 200 mg/kg, as well as that of the *C. citrinus* extract. This similarity can be explained by the fact that the compound or compounds responsible for producing the effect, which are contained in the extract, reach saturation at 50 mg/kg or even at lower concentrations. This suggests that the responsible compound or compounds may be highly potent, achieving the maximum effect even at the lowest concentration tested (50 mg/kg). However, further studies are needed to establish the therapeutic dosage range of phytosomes.

The *C. citrinus* phytosomes offer improved stability and solubility compared to *C. citrinus* extract due to their phosphatidylcholine formulation, which organizes the phytochemicals based on their functional groups. This arrangement increases the retention time of the phytosomes in the gastrointestinal tract, leading to improved bioavailability. Additionally, the phytosomes’ structure allows for better absorption of polar compounds and provides a hydrophobic bilayer for non-polar compounds. This contrasts with the low bioavailability and absorption of phenolic and flavonoid compounds in *C. citrinus* extract. Also, the major compounds found in *C. citrinus* extract are 1,8 cineole, limonene, and α-terpineol, which have high volatility and low bioavailability when administered orally.

This study also revealed that the liver is the first organ to be damaged during a high-fat-fructose diet. Previous studies have reported that a fructose diet is more obesogenic than a sucrose diet [32]. Additionally, research on high-fat diets has shown that they are sufficient to induce an increase in reactive oxygen species (ROS) and inflammatory status [33] or to cause a decrease in the activities of superoxide dismutase (SOD), catalase (CAT), and glutathione peroxidase (GPx) [34,35]. Other studies have reported that high-fat-sugar diets increase pro-inflammatory proteins more than high-fat or high-sugar diets [36].

Due to its heightened sweetening power, high fructose has been used instead of cane sugar in foods. However, this sugar is linked to significant health concerns, including the rise in obesity and its associated conditions. Fructolysis primarily occurs in the liver, increasing the synthesis of fatty acids, triglycerides, uric acid, and oxidative stress [37]. Several studies have indicated that a high fructose diet can result in metabolic syndrome and hyperinsulinemia [38]. Comparing results across studies can be challenging due to variations in fat concentration and type, sugar form (liquid or solid), and administration duration. Studies [38,39] have shown that gut microbiota and the production of advanced glycation end products are influenced by whether fructose is consumed in solid or liquid form. Furthermore, Collotta et al. [40] demonstrated that administering fructose in liquid form led to elevated levels of pro-inflammatory cytokines (TNF-α and IL-6).

In a study by García-Beltrán et al. [41], it was observed that levels of catalase (CAT), superoxide dismutase (SOD), glutathione S-transferase (GST), glutathione peroxidase (GPx), and quinone reductase (QR) activities were similar to those of the control group after 13 weeks. However, after 21 weeks, all the enzyme activities decreased compared to the control group. In contrast, our study found decreased SOD and CAT levels and increased GPx and GST levels in the liver and heart of rats fed a high-fat diet (HFD). Additionally, CAT and GST activities were reduced in the hearts of rats fed the HFD for 15 weeks. While both diets contain high levels of fat and fructose, the differences in rodent types and fructose administration methods may explain the variation. Additionally, GPx is a primary defense antioxidant enzyme, while GST is a detoxification enzyme combating toxic compounds. Both enzymes play a crucial role in maintaining GSH homeostasis [42,43]. Our study found that the rise in GPx and GST activities directly correlated with the decreased GSH levels in the liver and heart. GSH, acting as a reducing agent, serves numerous functions, including being a substrate for GPx and GST enzymes and providing protection against ROS. Based on our research, a high-fat-fructose diet (HFD) may reduce GSH levels and raise MDA, HEN, and AOPP levels in tissues. This could be because of increased free radical generation, resulting in oxidative stress in the tissues.

Our results contrast those reported by Norman et al. [44], who observed reductions in GPx, GST, and PON1 activities in the livers and hearts of rats fed a diet containing 46% fat and 24% sucrose. In contrast, our study involved a diet comprising 30% fat and 25% fructose. Our study demonstrated a notable increase in PON1 activity in the livers and hearts of rats treated with HFD. PON1 is recognized as an antioxidant enzyme due to its protective effects in the serum, being bound to HDL and preventing the oxidation of LDL [45]. Malondialdehyde (MDA) and hydroxynonenal (HNE) are the primary products of lipid peroxidation, known for their reactivity towards proteins and DNA, causing damage to these biomolecules [46]. Additionally, these products can impact the recruitment of cytokines such as TNF-α, IL-1β, IL-6, and COX-2. Two types of COX enzymes have been identified: COX-1, a constitutive enzyme found in most tissues, and COX-2, an inducible enzyme expressed during inflammatory processes [47]. According to Silva Santi et al. [48], a high-carbohydrate diet is associated with higher levels of inflammatory markers than a high-fat diet. Combining a high-fat and high-sugar diet also leads to more significant oxidative stress than a high-fat or high-sugar diet alone [49]. Furthermore, Almasri et al. [50] found that a diet containing 20% fat and 25% fructose increased inflammatory markers in the liver and skeletal muscle of rats

In our study, we observed an inflammatory process occurring in the group that was fed a high-fat-fructose diet. This was accompanied by increased cyclooxygenase activity and high adipose tissue deposition in this group (Table 1). The adipose tissue produced prostaglandins, which are known to be involved in the inflammatory process [51]. In contrast, the other groups maintained a low COX-2 activity similar to the control group, indicating that the extracts and phytosomes of *C. citrinus* could potentially inhibit this enzyme and prevent the inflammatory process. Limonene, one of the main compounds in *C. citrinus*, has been reported to reduce adipose tissue [31]. Lipoxygenases (LOXs) are enzymes involved in producing leukotrienes (LTs). LTs are mediators that impact chemotaxis, vascular function, fluid balance, immunity, and pain responses and are also part of the body’s inflammatory cascade [52]. The isoforms of LOX (5-LOX, 12-LOX, and 15-LOX) are present in the neuroinflammation process. The activity of LOX is regulated during oxidative stress [53]. In a study by Rudrapal et al. [54], it was reported that certain Indian spices can inhibit COXs and platelet activities, indicating their potential as anti-inflammatory agents. Our study found that the extract and phytosomes of *C. citrinus* effectively reduced COX-2 and platelet activities. In a previous study, we observed that *C. citrinus* only inhibited COX-2 while activating COX-1 [13]. These findings suggest that *C. citrinus* could be utilized as an anti-inflammatory agent due to its dual action against COX-2 and platelet activities.

Myeloperoxidase (MPO) is primarily found in neutrophils and serves as a bactericidal agent by reacting with chlorine ions (Cl^−^) and hydrogen peroxide (H_2_O_2_) to produce hypochlorous acid (HOCl). However, numerous reports suggest its involvement in various diseases due to its ability to produce reactive products such as chloramines, tyrosine radicals, and nitrogen dioxide. Additionally, it is considered a marker of inflammation [55]. Once again, the group that adhered to the high-fat-fructose diet exhibited an increase in MPO. Mazzoli et al. [56] observed that diets rich in fat and fructose are linked to enhanced oxidative stress in the liver, resulting in increased MPO activity. Furthermore, Lasker et al. [57] demonstrated that MPO activity rises in the livers of rats fed a high-fat diet for eight weeks. Van Leeuwen et al. [58] showed that MPO activity in the plasma initially increased in mice fed a high-fat diet but decreased after prolonged consumption. Our study revealed elevated MPO activity in the livers of rats fed a high-fat-fructose diet, while the same group exhibited low MPO activity in their hearts. These findings align with a report by Brennan et al. [59] indicating a protective role of MPO in atherosclerosis.

Xanthine oxidoreductase (XOR) plays a crucial role in purine catabolism, functioning as either a dehydrogenase (XDH) or an oxidase (XO). It is extensively present in the liver and intestines, generating the superoxide anion implicated in various inflammatory processes, including tissue damage and heart failure [60]. The liver exhibits the highest expression of XOR in its dehydrogenase activity. Under pathological conditions such as oxidative stress, the enzyme is an oxidant (XO) [61]. Our findings indicate a decrease in XO activity in the livers of rats fed a high-fat-fructose diet. The same group exhibited increased XO activity in the heart, possibly attributed to lard and vegetable shortening as fats, along with administering fructose in the food rather than in liquid form. Mastrocola et al. [39] reported varying results based on the method of fructose administration.

Orlistat is a medication used to treat obesity by inhibiting gastric and pancreatic lipase, preventing dietary triacylglycerol absorption. Recent studies have also demonstrated its antioxidant properties. Our study observed this antioxidant capacity in the liver and heart of rats fed a high-fat-fructose diet. This finding is consistent with the results presented by Hamza and Alsolami [62] and Othman et al. [63,64], who reported anti-atherogenic and antioxidant properties and regulated Nrf2 expression. Our study revealed a significant increase in glutathione-S-transferase activity in the hearts of the rats in the orlistat group compared to all other groups. Our study administered orlistat for 15 weeks, which is longer than most other studies. This could explain the result. This prolonged use of orlistat may lead to the generation of harmful compounds. The detoxification enzyme GST aids in transforming these compounds into GSH conjugates for elimination. Furthermore, overexpression of GST is detected in certain medical conditions [43].

The study revealed that orlistat positively impacts pro-inflammatory enzymes such as MPO, XO, COX-2, and LOX-5, as well as the levels of AOPP and HNE, which serve as biomarkers of oxidative stress. It has been reported that 1,8-cineole, the major compound in *C. citrinus*, increases the nuclear factor erythroid 2-related factor 2 (Nrf2) [65]. Additionally, 1,8-cineole, limonene, and α-terpineol reduce TNF-α, IL-6, and leptin levels [31].

The utilization of both the extract and phytosomes of *C. citrinus* in this study serves to validate its protective and anti-inflammatory properties, which can be attributed to the composition of its major phytoconstituents., 1,8-cineole, limonene, and α-terpineol [11,31]. The extract contains gallic acid, ellagic acid, p-coumaric acid, and phloroglucinol, which exhibit various biological activities such as antibacterial, antioxidant, anti-inflammatory, and antidiabetic properties [12]. These compounds work together to promote anti-inflammatory activity in rats fed a high-fat-fructose diet. Based on the study, it was found that the extract and phytosomes of *C. citrinus* were effective in reducing the levels of MPO, 5-LOX, and COX-2. This indicates that *C. citrinus* may help decrease the production of chlorinated products and the infiltration of immune cells in the tissues. Additionally, it was observed that it also lowers the activity of COX-2, resulting in reduced levels of PGE2, which is attributed to the presence of limonene, 1.8-cineole, and α-terpineol [66]. Limonene diminishes inflammation by decreasing the activity of 5-LOX and lowering the levels of LTB4, thereby preventing the inflammatory process [67]. Additionally, both limonene and terpineol demonstrate anti-inflammatory effects by decreasing the levels of pro-inflammatory cytokines such as TNF, IL-6, leptin, and AOPP in a colitis induction model [68]. Limonene reduces the production of ROS and RNS by increasing the activity of the antioxidant defense system in cells of diabetic rats and an inflammatory state [67]. It also promotes a reduction in MDA [69].

**Figure 6:**
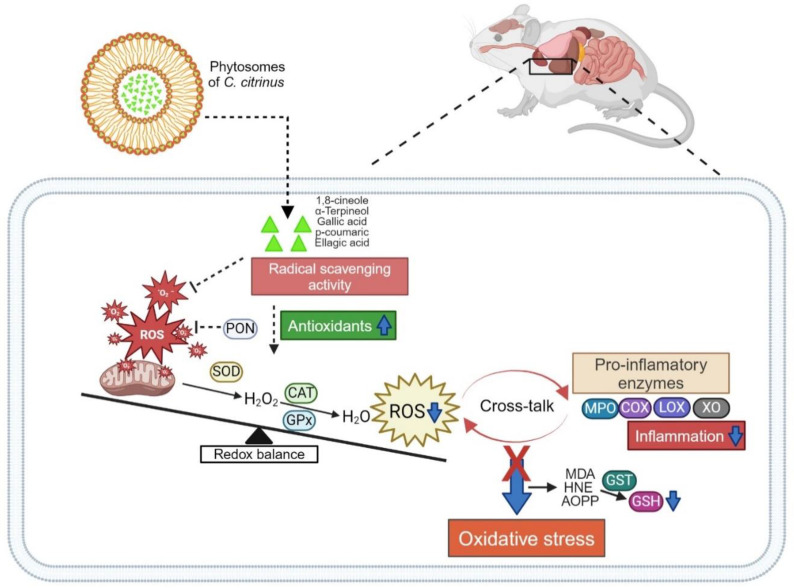
The proposed mechanism of action of *C. citrinus* phytosomes on oxidative stress and inflammation in rats induced by a high fat-fructose diet involves terpenes and phenolic acids. These components help reduce the increase in reactive oxygen species (ROS) by enhancing the activity of antioxidant and anti-inflammatory enzymes through enzyme induction and by lowering the biomarkers of oxidative stress. This information was created with BioRender.com.

In our study, we observed a significant increase in the levels of biomarkers of oxidative stress, such as MDA, HNE (indicating lipid peroxidation), and AOPP (indicating protein oxidation), in the livers and hearts of rats fed a high-fat diet. However, the administration of *C. citrinus* extract and phytosomes of *C. citrinus* led to a reduction in these levels compared to the control group, suggesting that *C. citrinus* may effectively scavenge free radicals. The results align with the functions of antioxidant enzymes. An imbalance in the antioxidant system and the generation of reactive oxygen species led to oxidative stress, which is associated with various disorders in obesity. Thus, treatment with compounds possessing higher antioxidant capacity may help mitigate the issues related to this condition (see Figure 6). It is essential to carefully consider the dosage and bioavailability of compounds intended for use as therapeutic agents. The present study observed that the lowest concentration of the *C. citrinus* phytosomes demonstrated a similar pattern to the higher concentration, thereby underscoring its significance. In the context of future studies, it is essential to consider using an additional analytical technique, such as Western blot or QPCR, to enhance further the understanding of the potential mechanism of action of phytosomes.

## 5. Conclusions

Phytosomes extracted from *C. citrinus* demonstrate the capacity to mitigate risk factors associated with oxidative stress, diminish the inflammatory process, and enhance the activities of antioxidant enzymes. Notably, even at low doses, dietary supplementation with phytosomes effectively averted the harmful effects of high-fat-fructose consumption.

## Figures and Tables

**Table 1:** Effects of supplementation of *Callistemon citrinus* extract and phytosomes on body weight gain and tissues in rats fed a high-fat-fructose diet.

Group	Body Weight Gain (g)	Adipose tissue (g)	Liver (g)	Heart (g)	Kidney (g)	Stomach (g)
C	178.80 ± 27.28 ^b^	12.01 ± 2.70 ^c^	12.77 ± 0.61 ^a^	1.07 ± 0.12 ^a^	3.00 ± 0.10 ^a^	1.85 ± 0.09 ^a^
C + V	170.20 ± 13.19 ^b^	12.12 ± 2.72 ^c^	13.42 ± 0.79 ^a^	1.37 ± 0.12 ^a^	2.88 ± 0.10 ^a^	1.85 ± 0.12 ^a^
C + *C. c* (200 mg/kg)	179.40 ± 20.42 ^b^	11.20 ± 2.70 ^c^	13.77 ± 0.61 ^a^	1.39 ± 0.14 ^a^	3.03 ± 0.11 ^a^	1.96 ± 0.10 ^a^
HFD	220.50 ± 13.48 ^a^	49.21 ± 3.02 ^a^	13.30 ± 0.68 ^a^	1.26 ± 0.12 ^a^	2.84 ± 0.08 ^a^	1.88 ± 0.09 ^a^
HFD + Orl (5 mg/kg)	180.75 ± 16.54 ^b^	27.12 ± 3.02 ^b^	14.65 ± 0.68 ^a^	1.27 ± 0.14 ^a^	2.81 ± 0.10 ^a^	1.86 ± 0.12 ^a^
HFD + *C. c* (250 mg/kg)	168.83 ± 12.47 ^b^	14.80 ± 3.49 ^bc^	12.26 ± 0.79 ^a^	1.30 ± 0.12 ^a^	2.75 ± 0.10 ^a^	2.01 ± 0.12 ^a^
HFD + P (50 mg/kg)	165.40 ± 29.97 ^b^	23.3 ± 3.49 ^bc^	12.00 ± 0.79 ^a^	1.30 ± 0.14 ^a^	2.72 ± 0.10 ^a^	1.90 ± 0.12 ^a^
HFD + P (100 mg/kg)	169.60 ± 28.04 ^b^	20.30 ± 3.49 ^bc^	12.14 ± 0.68 ^a^	1.25 ± 0.14 ^a^	2.64 ± 0.11 ^a^	1.81 ± 0.10 ^a^
HFD + P (200 mg/kg)	188.80 ± 16.91 ^b^	21.17 ± 3.49 ^bc^	12.97 ± 0.61 ^a^	1.26 ± 0.12 ^a^	2.69 ± 0.10 ^a^	1.99 ± 0.12 ^a^

All values expressed as mean ± SEM (*n* = 6; values statistically different ^(a, b, c)^ among groups (*p* ≤ 0.05) according to the Tukey test.

**Table 2:** Biomarkers of oxidative stress in the liver and heart in the control and experimental groups.

Biomarkers Oxidative Stress in Treatments					
Group	Organ	GSH (mM GSH/g Tissue)	MDA (nmol/mg Protein)	HNE (nmol/mg Protein)	AOPP (nmol/mg Protein)
C	Liver	8.72 ± 1.92 ^a^	0.41 ± 0.09 ^b^	0.35 ± 0.16 ^b^	8.13 ± 0.95 ^ab^
	Heart	17.34 ± 2.10 ^a^	0.25 ± 0.025 ^b^	0.18 ± 0.015 ^b^	8.97 ± 2.09 ^b^
C + V	Liver	6. 23 ± 1.63 ^a^	0.55 ± 0.07 ^b^	0.73 ± 0.31 ^b^	9.91 ± 1.95 ^ab^
	Heart	18.28 ± 0.44 ^a^	0.25 ± 0.016 ^b^	0.26 ± 0.053 ^b^	8.55 ± 1.09 ^b^
C + *C. c* (200 mg/kg)	Liver	6.55 ± 1.63 ^a^	0.38 ± 0.07 ^b^	0.57 ± 0.31 ^b^	6.32 ± 2.95 ^b^
	Heart	16.23 ± 0.88 ^ab^	0.24 ± 0.012 ^b^	0.22 ± 0.043 ^b^	9.62 ± 0.85 ^b^
HFD	Liver	2.08 ± 0.92 ^b^	0.86 ± 0.09 ^a^	2.55 ± 0.31 ^a^	13.97 ± 2.95 ^a^
	Heart	13.67 ± 0.88 ^b^	0.41 ± 0.018 ^a^	0.60 ± 0.018 ^a^	17.18 ± 2.09 ^a^
HFD + Orl (5 mg/kg)	Liver	6.17 ± 0.92 ^a^	0.55 ± 0.07 ^b^	1.17 ± 0.03 ^ab^	10.70 ± 2.15 ^ab^
	Heart	16.86 ± 0.57 ^ab^	0.30 ± 0.016 ^b^	0.13 ± 0.044 ^b^	12.52 ± 0.95 ^ab^
HFD + *C. c* (250 mg/kg)	Liver	6.85 ± 2.63 ^a^	0.58 ± 0.09 ^b^	0.87 ± 0.21 ^ab^	7.55 ± 1.95 ^b^
	Heart	15.14 ± 0.88 ^ab^	0.23 ± 0.030 ^b^	0.24 ± 0.020 ^b^	10.10 ± 1.10 ^b^
HFD + P (50 mg/kg)	Liver	6.36 ± 1.63 ^a^	0.41 ± 0.08 ^b^	1.20 ± 0.26 ^ab^	8.89 ± 0.95 ^ab^
	Heart	14.91 ± 0.46 ^ab^	0.28 ± 0.016 ^b^	0.26 ± 0.051 ^b^	11.53 ± 1.09 ^b^
HFD + P (100 mg/kg)	Liver	6.23 ± 0.92 ^a^	0.43 ± 0.02 ^b^	1.02 ± 0.31 ^ab^	7.54 ± 1.95 ^ab^
	Heart	15.92 ± 0.88 ^ab^	0.27 ± 0.012 ^b^	0.18 ± 0.043 ^b^	9.63 ± 1.09 ^b^
HFD + P (200 mg/kg)	Liver	6.09 ± 1.63 ^a^	0.44 ± 0.07 ^b^	0.33 ± 0.14 ^b^	6.22 ± 0.47 ^b^
	Heart	14.53 ± 1.88 ^ab^	0.21 ± 0.016 ^b^	0.18 ± 0.056 ^b^	9.14 ± 0.95 ^b^

All values expressed as mean ± SEM (*n* = 6; values statistically different ^(a, b)^ among groups (*p* ≤ 0.05) according to Tukey’s test.

## Data Availability

The original contributions presented in the study are included in the article further inquiries can be directed to the corresponding author.
